# Machine Learning Algorithms Associate Case Numbers with SARS-CoV-2 Variants Rather Than with Impactful Mutations

**DOI:** 10.3390/v15061226

**Published:** 2023-05-24

**Authors:** Matthieu Vilain, Stéphane Aris-Brosou

**Affiliations:** 1 Department of Biology, University of Ottawa, Ottawa, ON K1N 6N5, Canada; 2 Department of Mathematics and Statistics, University of Ottawa, Ottawa, ON K1N 6N5, Canada; sarisbro@uottawa.ca

**Keywords:** machine learning, random forest, feedforward neural network, COVID-19, SHapley Additive exPlanation (SHAP), bias

## Abstract

During the SARS-CoV-2 pandemic, much effort has been geared towards creating models to predict case numbers. These models typically rely on epidemiological data, and as such overlook viral genomic information, which could be assumed to improve predictions, as different variants show varying levels of virulence. To test this hypothesis, we implemented simple models to predict future case numbers based on the genomic sequences of the Alpha and Delta variants, which were co-circulating in Texas and Minnesota early during the pandemic. Sequences were encoded, matched with case numbers at a future time based on collection date, and used to train two algorithms: one based on random forests and one based on a feed-forward neural network. While prediction accuracies were ≥93%, explainability analyses showed that the models were not associating case numbers with mutations known to have an impact on virulence, but with individual variants. This work highlights the necessity of gaining a better understanding of the data used for training and of conducting explainability analysis to assess whether model predictions are misleading.

## 1. Introduction

Since the publication of the seminal work of the late Sir Robert May [1], most of modern epidemiology aims at predicting the severity of viral outbreaks based on the number of individuals who are susceptible, infected, and recovered (or dead) in a population—that is, based on epidemiological data, as has been the case, for instance, during the COVID-19 pandemic, caused by the SARS-CoV-2 virus [2]. Complementary approaches have nonetheless resorted to machine learning (ML) to improve predictions, but these applications mainly focused on the same kind of epidemiological data [3,4,5,6,7] or on image processing to diagnose the disease [8].

However, these approaches all have in common that the viral genome is seldom part of the equation, which is odd because the dynamics of a viral outbreak are governed not just by contacts among hosts (people), but also by the viral genome and the mutations that may be changing its virulence, transmissibility, and ultimately, viral fitness, i.e., affecting the severity of an outbreak. Such an accumulation of mutations has occurred during the COVID-19 pandemic, as multiple variants and sub-variants were co-circulating in communities [9] with specific transmission rates. For instance, the Alpha variant has been reported to be 40 to 90% more transmissible than previous variants [10]. To date, we are not aware of any attempt to use ML for predicting case numbers of an outbreak solely based on genomic data.

Here, building on recent work that aimed at predicting the genetic determinants of complex phenotypes from whole-genome sequence data using simple ML algorithms [11], we hypothesized that the severity of COVID-19, and more specifically its case numbers, can be predicted without any a priori assumptions or epidemiological data, using simple ML models such as Random Forest (RF) and Feed-Forward Neural Network (FFNN) trained solely on genomic data. After training these models on publicly available data, their performances were compared and an explainability analysis was conducted. This led us to show that despite high prediction accuracy, the models actually learned to classify variants rather than identifying mutations that have a biological impact.

## 2. Materials and Methods

### 2.1. Data Retrieval

Epidemiological data from two US states, Minnesota (MN) and Texas (TX), were retrieved using the COVIDcast Dashboard. This tool allows for the real-time extraction of statistics describing the COVID-19 pandemic as provided by the Johns Hopkins University and USAFacts [12]. We focused on these two states because of their differences in population size, population flow (Dallas is one of the biggest airports in the US), and restriction guidelines during the pandemic. We specifically retrieved the average number of new COVID-19 cases per 100,000 individuals over a 7-day window (confirmed_7day_incidence_prop; windows end at focal days retrieved) for every single day for which we extracted genomic data, as detailed below. The mean (±one standard deviation) case numbers that were retrieved were similar across the three datasets analyzed: 17.49 (±17.67) for MN, 26.54 (±21.32) for TX, and 22.12 (±20.13) for the combined dataset (see Table S1 for details).

Complete viral genomes were downloaded from GISAID [13], as we aimed to predict COVID-19 cases irrespective of the circulating variants. We focused on a time window when both the Alpha and Delta variants were present, early in the pandemic, and retrieved genomic data from the two targeted US states, MN and TX. The downloaded genomes were checked for quality to keep only genomes ≥29,000 nucleotides in length, of high coverage (≤1% unidentified nucleotides), and without any uncertain nucleotides (only T, C, A, or G) or incomplete collection date (Y/M/D). All the Alpha sequences were collected between 1 April and 7 May 2021, as this was the window when the highest number of Alpha sequences were available, and were approximately equally distributed between the two states. The Delta sequences used were collected in August 2021, when the highest number of sequences were available. As more sequences were available from TX than from MN, sequence data from TX were randomly subsampled to match the number of sequences from MN to avoid any imbalance.

Sequences from both states were merged and aligned with MAFFT v7.471 [14], trimmed with TrimAl v1.4 [15] (with default settings) to remove poorly aligned regions, and sequences containing indels were removed. The numbers of sequences used henceforth were 3585 for MN (Alpha: 2382; Delta: 1203) and 3741 for TX (Alpha: 1560; Delta: 2181). Given their collection dates, each sequence could either be matched to the case numbers from the corresponding state on this very same day or to case numbers at a later date. This time lag, denoted below as *days ahead* or *ℓ*, allowed us to test the accuracy of the models for predictions at different time ranges.

### 2.2. Encoding Genetic Data

Genomic sequences were then transformed (*embedded*) into numeric vectors to be amenable to downstream ML analyses. For this, sequences were split into non-overlapping contiguous *k*-mers (words of length *k*) and embedded with the Term-Frequency Inverse-Document-Frequency (TF-IDF) algorithm [16]. This algorithm starts by mapping the set of all unique *k*-mers across every documents (the aligned sequences). Then, for each document (sequence) and each word (*k*-mer) in the vocabulary, the term frequency (TF: number of occurrences of each word) in the current document is calculated and weighted by the inverse document frequency ($IDF=\log_{10}\left(\frac{n}{df(t)}\right)+1$, where *n* is the number of documents and df(t) is the document frequency for this term), so that TF-IDF =TF×IDF. This algorithm was implemented using python v3.7 and the TF-IDFVectorizer class from the Scikit v0.24.2 package [17].

### 2.3. ML Training and Testing

The performance of select ML algorithms, including Random Forest (RF), Feed-Forward Neural Network (FFNN), Convolutional Neural Network, and Convolutional Long Short-Term Memory Neural Network, was then assessed, but only RF and FFNN were retained for follow-up analyses. This was done because all the models tested had similar performance, while RF and FFNN were simpler and thus more amenable to the explainability analyses described below. The RF model was implemented via the class RandomForestRegressor from Sklearn v0.24.2 [17], and FFNN was implemented through Keras and Tensorflow v2.7 [18].

Both models were trained and tested following the same workflow. First, we kept all model parameters to their default values and tuned two parameters, *k*-mer length and days ahead *ℓ*, to optimize accuracy. These two parameters are critical, *k*-mer length representing the complexity of the models (longer *k*-mers lead to more words, and hence more features), and days ahead *ℓ* standing for the incubation period [19]. Models were trained and tested using five-fold cross-validation, and accuracies were then averaged over these five replicates. We tested every combination of *k*-mer lengths for k∈(2,8) by increments of 1, and of days ahead for ℓ∈(7,73) by increments of 2.

Once the combination (*k*-mers, *ℓ*) with the highest average accuracy was determined, model hyper-parameters were fine-tuned with Keras-Tuner [20]. For this, the data were randomly split into training (60%), validation (15%), and testing subsets (25%) with the train_test_split function from Sklearn, the training set being used to train the model, the validation one to assess the model after each epoch, and the testing one to test the accuracy of our model once it was fully tuned. Keras-Tuner determined the optimal value for each hyper-parameter based on 300 runs using a Bayesian optimization (BayesianOptimization class). The Mean Absolute Percentage Error (MAPE) was used to assess accuracy on the fully tuned model. To gauge the efficiency of the tuning steps and the robustness of our predictions, a second round of training and testing was performed. For each model (RF and FFNN) and each dataset (MN, TX, MN + TX), the hyper-parameters from the previous step were used, and both the length of *k*-mers and day ahead *ℓ* were re-optimized.

Finally, accuracies under RF and FFNN were compared for each state. For this, sequences were repeatedly split into training, validation, and testing sets using different random seeds, so that different sets were obtained under each repeat. This was carried out 16 times, and accuracies were compared with a *t*-test.

### 2.4. Explaining Models Output

To better understand what models are learning, the distribution of case numbers for every lag period *ℓ* tested was extracted and compared. For the lag that gave the best accuracy, the distribution of the number of sequences matched with case counts according to their variant type (either Alpha or Delta) was then plotted.

To investigate how RF and FFNN are making predictions, a SHapley Additive exPlanation (SHAP) analysis [21] was conducted to analyzed which model inputs (i.e., *k*-mers) were the most important to predict case numbers. The package SHAP v0.41.0 uses a game-theoretic approach to explain the output of any ML model. Here, all training examples and trained models were passed to SHAP’s DeepExplainer function. SHAP uses the training example as background samples to compute the importance values of each *k*-mer for every sequence in our test dataset. These values represent the extent to which a *k*-mer shifts the predicted case numbers, either upwards or downwards.

### 2.5. Mapping k-mers Back to the Genome

The 30 most important *k*-mers were then mapped to their genomic positions (i) to test if these *k*-mers were shared between variants or not, and (ii) to characterize the top *k*-mers from a functional point of view. For this, MAFFT was used to re-align the sequences with the SARS-CoV-2 reference genome (NC_045512), hence identifying the genomic position of each *k*-mer. Each nucleotide within each *k*-mer was then compared against the nucleotide of the reference sequence at the same position. If the nucleotides were different, this mutation was looked up in the National Genomics Data Center SARS-CoV-2 variation database (https://ngdc.cncb.ac.cn/ncov/variation/annotation, accessed on 1 February 2023), which contains information on the type, impact, and evidence level for each known mutation. Indels were ignored, since we removed sequences containing any from our alignments. The impact of mutations on SHAP importance scores was tested with a Kruskal–Wallis analysis since the assumption of normality was not met. The mutation data were limited to those with the highest level of evidence (level V), unless otherwise stated.

With this, it could be tested if the models were giving more importance to *k*-mers that were only existing in both variants, or only in one of them. The number of Alpha and Delta sequences for each mutation was recorded, and an index to determine if the mutation was present in both variants was computed as $\left|\left(N_{a}/T_{a}\right)-\left(N_{d}/T_{d}\right)\right|$, where $N_{a}$ and $N_{d}$ are the number of Alpha and Delta sequences which have the mutation, and $T_{a}$ and $T_{d}$ are the total number of sequences of Alpha and Delta variants in the dataset. The absolute value was used to obtain a positive index quantifying the restriction of mutations to a particular variant; a larger value indicating a mutation is almost exclusively found only in one of the variants.

## 3. Results

### 3.1. Both Days Ahead and k-mer Lengths Affect Accuracy

**Figure 1:**
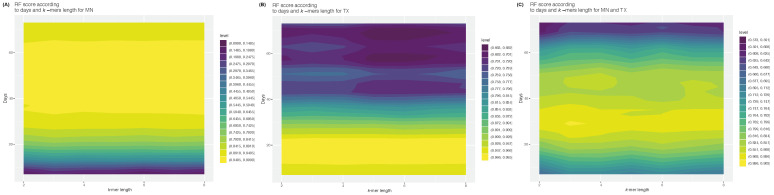
Accuracy for RF as a function of *k*-mer length and number of days ahead on the three datasets: (**A**) MN; (**B**) TX; (**C**) both states. Color scales range from low (colder hues) to high (warmer hues).

To gauge how *k* and *ℓ* affect the accuracy of the predictions, model accuracies were plotted under both models, RF and FFNN. For RF, the results for each state taken independently suggested that *k*-mer length does not have a major impact on the accuracy of predictions (Figure 1). However, accuracy varied with *ℓ*, with an optimal window between 35 and 63 days for MN (Figure 1A) and 12 and 22 days for TX (Figure 1B). While this lag approximately corresponds to the two-week incubation period in TX, the longer lag in MN could suggest a longer intrinsic generation time (delay between the infection of a first and second case) or a longer diagnostic delay (delay between infection and diagnosis). As expected, when data from both states were merged, intermediate results were obtained, with higher accuracies around k=3 and ℓ∈(25, 38). Accuracies on the MN+TX dataset were also lower than when each state was analyzed individually, suggesting that RF performs poorly when making predictions across heterogeneous populations (Figure 1C), even when trained on such heterogeneous populations.

**Figure 2:**
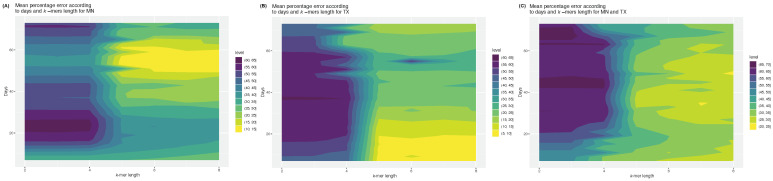
Accuracy for FFNN as a function of *k*-mer length and number of days ahead on the three datasets: (**A**) MN; (**B**) TX; (**C**) both states. Color scales range from low (colder hues) to high (warmer hues).

Likewise for FFNN, similar patterns were observed for days ahead, but under this more complex model, both *ℓ* and *k* affected accuracy, which increased sharply from k=5, or about two codons (six nucleotides; Figure 2). This was expected, as longer *k*-mers lead to a larger lexicon, hence to models including a larger number of features, and hence to a better performance.

### 3.2. Best Performances Are State-Specific

With those optimal combinations of model parameters for RF (MN: k=8, ℓ=51; TX: k=7, ℓ=17; both: k=3, ℓ=29) and FFNN (MN: k=7, ℓ=57; TX: k=6, ℓ=7; both: k=7, ℓ=35), the hyper-parameters of each model were fine-tuned. Surprisingly, for both RF (Table 1A) and FFNN (Table 1B), the optimal architecture for TX was simpler than for the two other datasets. This result is consistent with the unexpected results obtained for MN (Figure 1 and Figure 2), suggesting that this latter dataset, both from an epidemiological and a population point of view, is more complex than the one from TX.

Under these fine-tuned models, the best accuracies are shown in Table 1C. FFNN outperforms RF on the MN and TX + MN datasets ($P=2.20\times 10^{-16}$ and $P=9.98\times 10^{-14}$, respectively). However, the difference in accuracy was not significant for the TX dataset (P=0.146), and predictions were systematically worse for MN. While state-specific accuracies could be as high as 93.66%, performance dropped significantly when the data were aggregated over multiple states, dropping as low as 70%, in spite of the the dataset being larger. Further efforts should hence aim at modeling heterogeneous data.

### 3.3. Mapping k-mers Back to the Genome

**Figure 3:**
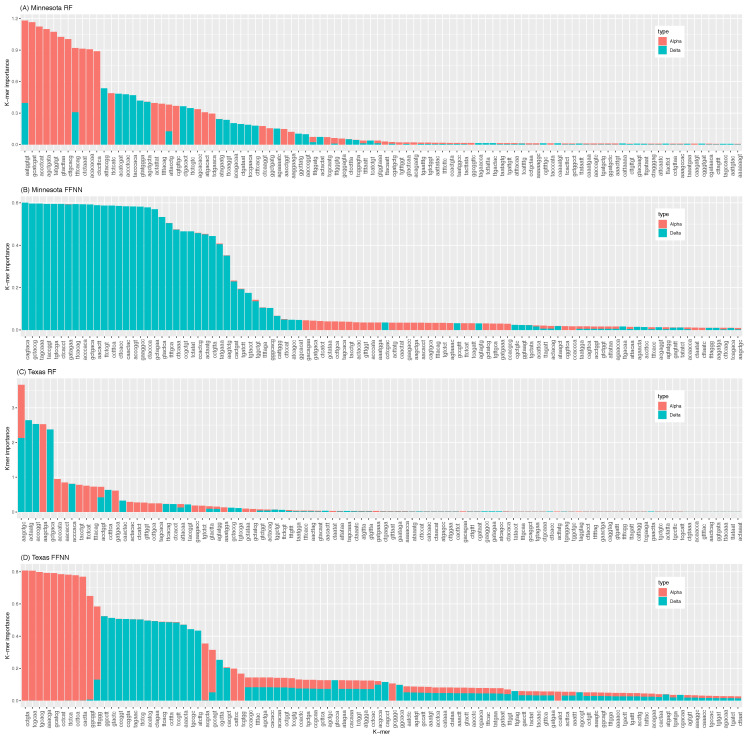
SHAP scores of the top 100 most important *k*-mers. Scores are shown for both the model and both datasets, ranked by decreasing value. Results for MN are shown for RF (**A**) and FFNN (**B**). Results for TX are shown for RF (**C**) and FFNN (**D**). The *k*-mers from the Alpha variant are shown in blue, while those from the Delta variant are in red.

To understand how the models were making predictions and if the most important mutations they identified were also the most relevant from a biological standpoint, an explainability analysis of model predictions was performed for the MN and TX datasets. The post-fine-tuning SHAP analysis showed that for FFNN in MN, the most important *k*-mers were almost exclusively from the Delta variant, while in TX, they were mostly from the Alpha variant, with a few from the Delta variant at lower importance values (Figure 3). This could be in part due to the imbalance between variants we have in our dataset. Indeed, MN has fewer Delta variants (Alpha: 2382; Delta: 1203), while TX has fewer Alpha variants (Alpha: 1560; Delta: 2181). However, it is unlikely that that imbalance would be responsible for this pattern, as for RF, the opposite trend was found (Figure 3).

**Figure 4:**
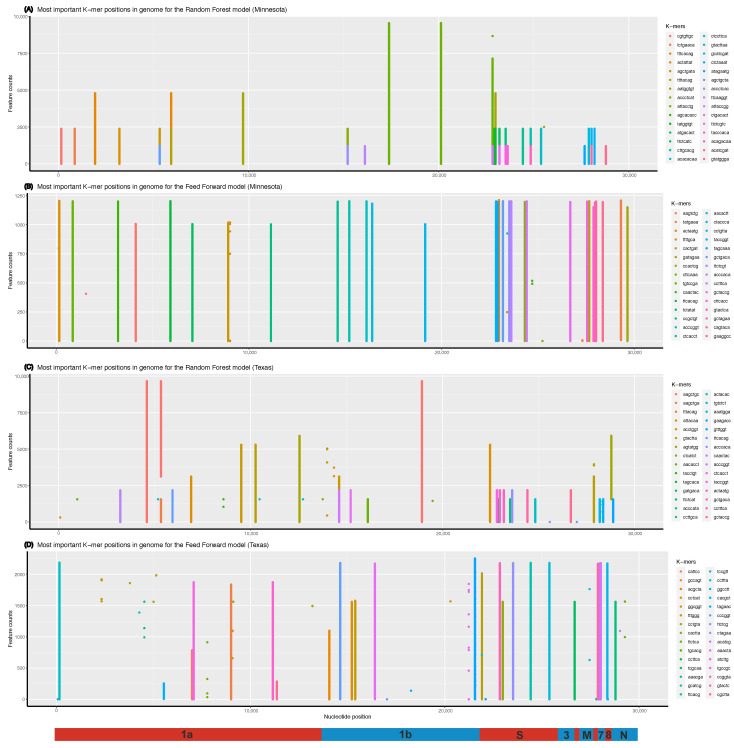
Distribution of the 30 most important *k*-mers for each model and each individual state along the SARS-CoV-2 reference genome (shown at the bottom of each column). Results for MN are shown for RF (**A**) and FFNN (**B**). Results for TX are shown for RF (**C**) and FFNN (**D**).

To understand the biological significance of these results, these *k*-mers were mapped back to the viral genome, limiting this analysis to the top 30 features for clarity. The spatial distribution of these *k*-mers shows that the most important ones were located between positions 22,000 and 25,000 bp (Figure 4), which corresponds to the genomic region encoding the spike protein. Other important *k*-mers were located in regions related to virulence, such as the non-structural protein 1b (between 14,000 and 16,000 bp), responsible for replication and transcription of the viral RNA [22].

**Figure 5:**
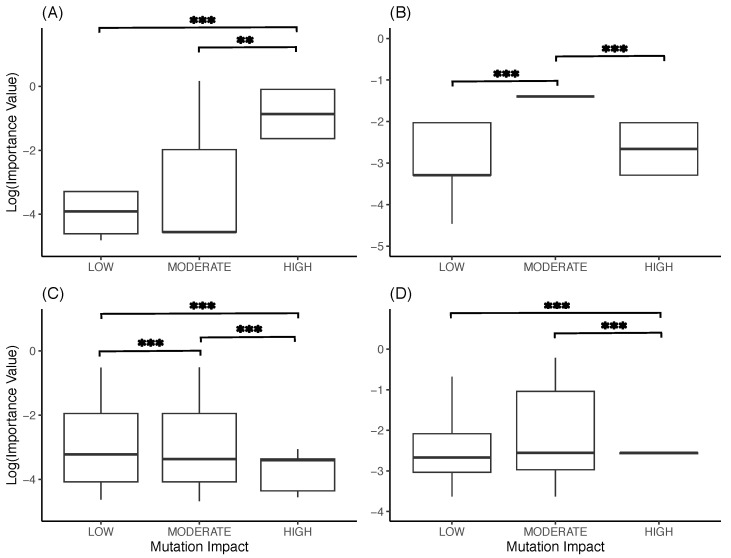
Boxplots of the importance score of *k*-mers according to the impact of mutations found in them. (**A**) RF model for Minnesota. (**B**) RF model for Texas. (**C**) FFNN model for Minnesota. (**D**) FFNN model for Texas. Significance was assessed with a Kruskal–Wallis test **: *p* < 0.01; *** *p* < 0.001.

However, the location of the most important *k*-mers in virulence genes does not imply their direct impact on case numbers, as it is possible that some of these *k*-mers were important because the models learned how these features differed between the two variants. To gauge this possibility, all the mutations at those *k*-mer positions were identified, and using the NGDC’s SARS-CoV-2 variation database, their phenotypic impact, graded as low, moderate, and high, was extracted. For both models and both states, there are highly significant differences in importance values across the three classes of phenotypic impact (RF MN: $P=8.15\times 10^{-13}$; RF TX: $P=2.20\times 10^{-16}$; FFNN MN: $P=2.20\times 10^{-16}$; FFNN TX: $P=2.20\times 10^{-16}$). If the results were biologically relevant, one would expect that the most important *k*-mers should also be those with the largest phenotypic impact. However, this is not the case (Figure 5), suggesting that the most important *k*-mers found to be predicting case numbers are not involved in virulence.

### 3.4. What the Models Learn

**Figure 6:**
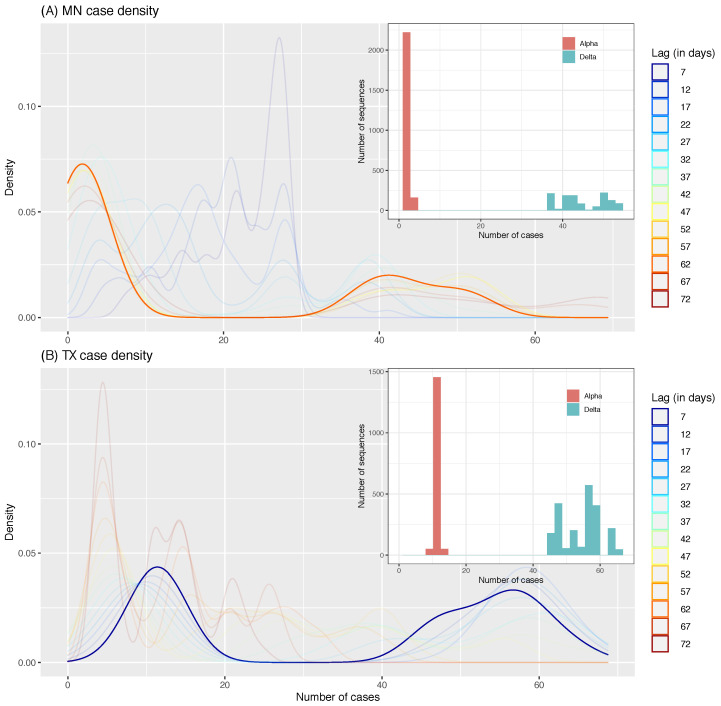
Density distribution of cases to predict in our dataset according to the lag. (**A**) is the dataset for Minnesota and (**B**) is for Texas. The warmer colors represent the distribution for longer *ℓ*, colder colors represent the distribution for shorter *ℓ*, and the solid line highlights the distribution for the lag that gave the best accuracy. Insets show the number of sequences of each variant matched to case numbers to predict for that solid line distribution.

To go further, the distribution of the number of cases was plotted for every lag (ℓ∈[7, 72] days) used when tuning the models, and the one returning the best accuracy was highlighted (bold line in Figure 6). For both datasets, this distribution at optimal lag is bimodal, and the corresponding distribution of variants is also bimodal (Figure 6, insets), thereby clearly showing that each peak in case numbers corresponds to a particular variant, Delta having systematically the highest number of cases. This shows that the models learned to associate each variant with case numbers by detecting mutations that are unique to each variant, rather than detecting those mutations that alter the transmissibility of the virus. This is further supported by the positive and significant correlation between mutations only found in one of the two variants and their associated importance score for every model/state combination (Table 2).

## 4. Discussion

Both the RF and FFNN models described here returned very accurate predictions, being as high as 93%. These results were unexpected, mostly because prior machine learning models attained similar results, but at the cost of implementing more complex approaches resorting to time series of case numbers [3]. However, comparing previous approaches with those described here may not be entirely fair, as we smoothed case numbers by taking their moving average over 7-day windows. This relatively large temporal window might have contributed to making our data “simpler” (less noisy), hence leading to high performance metrics. Other works, on the other hand, aimed at making predictions for the 50 most populous counties in the US using spatiotemporal data reported worse MAPE than our models, reaching accuracies no higher than 80% [23], as averaged for every 50 counties over their whole evaluation period, which is much longer than ours. Irrespective of this, as these prior studies relied solely on epidemiological data, our work demonstrates the value of considering genomic information when making predictions about case numbers. It can be predicted that combining epidemiological and genomic data can only lead to improved performance.

To better understand these performances, however, an explainability analysis was performed using SHAP, identifying which *k*-mers were the most important, and thereby finding that the most important *k*-mers were often only found in one of the two variants. Trying to gauge if these *k*-mers still had a biological interpretation, it was found that the most important *k*-mers were located in similar genomic regions, in particular in Non-Structural Protein (NSP) 12, in the Open Reading Frame (ORF) 1ab, in the spike protein, and in ORFs 7 and 8. These genomic regions are known to be functionally important for viral replication, transmissibility, and virulence; indeed, NSP 12 forms an essential complex with NSP 7 and NSP 8 for viral replication, the spike protein is crucial for cell entry, and ORFs 7 and 8 act as an immune evader/modulator [24]. However, the idea that the RF and FFNN models identified important mutations is not supported by the analysis of their phenotypic impact, as *k*-mers containing mutations with high impact did not have a higher importance than mutations with moderate or low impact (Figure 5).

Contrary to expectations, the significant correlation between mutations being only found in one variant and the *k*-mers importance score confirms that the models learned to tell variants apart when predicting case numbers. This is due to the unfortunate sampling of variants, where each state was in the midst of the Delta wave, which was itself responsible for most of the case numbers, while the Alpha variant was on the decline. While collecting genomic data, we focused too much on limiting class imbalance, finding dates for which we would have similar numbers of sequences for both variants in both states. We can observe this in Figure 6, where for both RF and FFNN, the models had a better accuracy when case numbers followed a bimodal distribution. This effectively creates a dataset where the models have to try to tell apart two time windows instead of using past information to try to predict future case numbers as in time series-based models. This also suggests that the optimal lags that we identified do not correspond to the incubation time of the virus. This is further supported by findings reporting incubation time ranging from 1 to 18 days, with a mean of 6.53 days [25]. While the optimal lag for TX was in that range, the one for MN, between 35 and 65 days, is way past this known incubation period.

Since the most important *k*-mers were unique to a given variant and located in genomic regions related to transmissibility/virulence, it is likely that the main differences between the two variants are in these regions, possibly because such regions are under selective pressure. Indeed, many sites were under selection in the SARS-CoV-2 wild type, especially in NSP 12 and the spike protein, while only three sites were under selection in ORF 7 and only one in ORF 8 [26]. More specifically, most mutations specific to the Alpha and Delta variants described previously were found in the datasets of mutations of each model/state combination linked to the important *k*-mers identified here [27].

## 5. Conclusions

During the COVID-19 pandemic, a substantial effort has been made to develop ML models to either predict case numbers from epidemiological data [3,4,5,6,7] or to classify SARS-CoV-2 sequences using genomic data [28,29,30,31], but in most situations, there was no attempt to explain the output of these models. With recent progress in model explainability, ML models are less and less considered as black boxes, and explaining them is especially important in epidemiology and biology. Indeed, understanding how the models make predictions is necessary to gauge the accuracies of said predictions, which is critical if the models are to be used by governing bodies or policymakers. Moreover, explaining model predictions can provide us with useful insights into the data used to train the model; here, for instance, explainability analysis allowed us to identify unique mutations and *k*-mers characterizing the Alpha and Delta variants, but this kind of analysis can also be used to identify biomarkers that predict the mortality of patients infected by SARS-CoV-2 [32]. Our work hence represents a call for being careful when collecting training data and for trying as much as possible to understand what is being learned by models when predicting case numbers. Finally, future work should assess the extent to which integrating genomic data with epidemiological data into ML models can improve case number predictions.

## Figures and Tables

**Table 1:** Hyper-parameters of RF and FFNN for all three datasets. (**A**) Optimal model architecture for RF and (**B**) for FFNN. (**C**) Final accuracies under each model and dataset.

(**A**)	**Random Forest**		
	**MN**	**TX**	**MN + TX**
Estimators	1960	1810	1960
Criterion	Absolute Error	Absolute Error	Squared Error
Depth	False	True	False
Maximum depth	N/A	233	N/A
Minimum sample split	2	78	2
Minimum sample leaf	1	1	1
Maximum features	Auto	Auto	Auto
(**B**)	**Feed-Forward Neural Network**		
	**MN**	**TX**	**MN + TX**
Number of layer	3	1	3
Activation function	ReLU	Softplus	ReLU
Dropout	False	False	False
Unit layer 1	512	512	224
Unit layer 2	512	N/A	8
Unit layer 3	152	N/A	512
Learning rate	0.0001	0.0203	0.0001
(**C**)	**Final Results**		
	**RF**	**FFNN**	
MN	87.82%	88.15%	
TX	91.25%	93.66%	
MN + TX	70.20%	75.32%	

N/A: not applicable.

**Table 2:** Effect of the presence of mutations in one variant or the other on importance scores. The intercept, effect of the presence of a mutation, and adjusted $R^{2}$ values of the linear regression between importance score and presence of mutation are presented for each model/state combination.

Variable	RF/MN	RF/TX	FFNN/MN	FFNN/TX
Intercept	0.1698 **	0.1039 **	0.1438 **	0.1481 **
Effect of a mutation	0.1762 **	0.3132 **	0.0686 *	0.1138 **
Adjusted $R^{2}$	0.0469	0.0698	0.0133	0.0296

* *p* < 0.05, ** *p* < 0.01.

## Data Availability

The code developed for this work is available from www.github.com/sarisbro/data/, accessed on 3 April 2023. All sequence data are available from gisaid.org/, accessed on 23 December 2021.

## Supplementary Materials

The following supporting information can be downloaded at: https://www.mdpi.com/article/10.3390/v15061226/s1, Table S1: Means and standard errors of the case numbers per 100,000 people for each lag period and each dataset.
