# Supplementary material for: Machine Learning Algorithms Associate Case Numbers with SARS-CoV-2 Variants Rather Than with Impactful Mutations
Source: Viruses. 2023 May 24;15(6):1226. doi: 10.3390/v15061226 (PMC10300801; doi:10.3390/v15061226)
Supplement: Supplementary file 1 [file viruses-15-01226-s001.zip › viruses-2354801-supplementary.pdf]

Table S1. Means and standard errors of the case numbers per 100,000 people for each lag period and each dataset.

Lag (in days)	MN		TX		Combined	
	mean	sd	mean	sd	mean	sd
7	21.91	5.95	36.55	21.70	29.39	17.64
12	19.59	6.68	37.44	22.94	28.70	19.24
17	18.00	8.68	**38.35**	**24.24**	28.39	20.99
22	17.08	11.40	37.49	24.36	27.50	21.70
27	16.11	13.44	35.21	23.70	25.87	21.59
32	15.35	15.53	33.26	23.58	**24.49**	**21.95**
37	15.38	17.57	29.79	22.10	22.74	21.27
42	16.64	19.95	26.80	20.62	21.83	20.92
47	17.22	21.43	23.05	17.03	20.20	19.53
52	**17.05**	**21.61**	19.21	13.51	18.15	17.97
57	16.29	20.65	16.67	11.79	16.48	16.72
62	16.15	20.29	14.28	9.35	15.20	15.71
67	18.10	23.08	12.58	7.62	15.28	17.26
72	20.06	24.80	10.94	6.22	15.40	18.48

Notes—MN: Minnesota; TX: Texas; sd: standard error; bold: optimal lag for each dataset.
